# Hypothermia Does Not Boost the Neuroprotection Promoted by Umbilical Cord Blood Cells in a Neonatal Hypoxia-Ischemia Rat Model

**DOI:** 10.3390/ijms24010257

**Published:** 2022-12-23

**Authors:** Inês Serrenho, Carla M. Cardoso, Mário Grãos, Alexandra Dinis, Bruno Manadas, Graça Baltazar

**Affiliations:** 1 Health Sciences Research Center (CICS-UBI), University of Beira Interior, 6201-506 Covilhã, Portugal; 2 CNC—Center for Neuroscience and Cell Biology, University of Coimbra, 3004-504 Coimbra, Portugal; 3 Crioestaminal (Stemlab S.A.), 3060-197 Cantanhede, Portugal; 4 Biocant, Technology Transfer Association, 3060-197 Cantanhede, Portugal; 5 Institute for Interdisciplinary Research, University of Coimbra (IIIUC), 3030-789 Coimbra, Portugal; 6 Pediatric Intensive Care Unit, Hospital Pediátrico, Centro Hospitalar e Universitário de Coimbra, 3004-561 Coimbra, Portugal; 7 Faculty of Health Sciences, University of Beira Interior, 6201-506 Covilhã, Portugal

**Keywords:** neonatal hypoxic-ischemic encephalopathy, umbilical cord blood cells, cell therapy, therapeutic hypothermia, neonatal brain injury

## Abstract

Neonatal hypoxic-ischemic encephalopathy (HIE) is one of the leading causes of death and long-term disability in the perinatal period. Currently, therapeutic hypothermia is the standard of care for this condition with modest efficacy and strict enrollment criteria. Therapy with umbilical cord blood cells (UCBC) has come forward as a strong candidate for the treatment of neonatal HIE, but no preclinical studies have yet compared the action of UCBC combined with hypothermia (HT) with the action of each therapy by itself. Thus, to evaluate the potential of each therapeutic approach, a hypoxic-ischemic brain lesion was induced in postnatal day ten rat pups; two hours later, HT was applied for 4 h; and 24, 48, and 72 h post-injury, UCBC were administered intravenously. The neonatal hypoxic-ischemic injury led to a brain lesion involving about 48% of the left hemisphere that was not improved by HT (36%) or UCBC alone (28%), but only with the combined therapies (25%; *p* = 0.0294). Moreover, a decrease in glial reactivity and improved functional outcomes were observed in both groups treated with UCBC. Overall, these results support UCBC as a successful therapeutic approach for HIE, even when treatment with therapeutic hypothermia is not possible.

## 1. Introduction

Neonatal hypoxic-ischemic encephalopathy (HIE) may result from acute events that decrease oxygenated blood flow to the neonate’s brain during the peripartum period. This condition is one of the leading causes of neonatal death and long-term disability worldwide, with 0.5 to 1 in 1000 term neonates being affected in developed countries [1]. Approximately 20% of the newborns who survive the initial insult develop moderate to severe long-term impairments, such as cerebral palsy [2]. Currently, therapeutic hypothermia beginning within 6 h after birth is the standard of care for term neonates with moderate to severe HIE [3]. However, therapeutic hypothermia lacks effectiveness in severe cases and cannot always be applied due to strict enrollment criteria [1]. Thus, the scientific community remains focused on uncovering adjuvant or alternative therapies that improve outcomes for these patients.

One of the therapeutic approaches being explored is the autologous administration of umbilical cord blood cells (UCBC). Umbilical cord blood is an excellent source of stem cells, such as hematopoietic stem cells and, in a lower proportion, mesenchymal stem cells (MSC), but also endothelial progenitor cells and immune cells [4]. Studies in newborns with HIE demonstrated that autologous administration of UCBC was safe and feasible, but until now, no neurodevelopmental follow-up results from these studies have been published to testify its efficacy [5,6]. Although several preclinical studies [7,8,9,10,11,12,13,14] demonstrated that systemic administration of UCBC after a hypoxic-ischemic (HI) event improved functional disabilities, reduced brain damage, and decreased glial reactivity (reviewed by [15]), few studies have been published assessing the combination of hypothermia (HT) with UCBC in in vivo models for HIE. To our knowledge, only three studies evaluated the combination of TH and stem cell therapy [16,17,18], and in one of these studies, the combination of HT with MSC was not effective and elicited an inflammatory response [18].

While preclinical studies demonstrated that the administration of UCBC can be a promising therapy for HIE, it is necessary to optimize protocols considering a possible clinical application, therefore evaluating the combined effect of HT with the administration of UCBC. This study was designed to be aligned with clinical applicability; namely, UCBC were administered by an intravenous route with a schedule feasible in clinical practice. The two main objectives of this study were to evaluate if UCBC therapy could reduce glial reactivity, which is a key player in the neuroinflammatory response after an HI insult in the developing brain, and weather each therapy by itself or combined could induce a significant reduction of brain lesion extension and promote the recovery of the functional deficits previously described in this model.

## 2. Results

### 2.1. Effect of Hypothermia and Umbilical Cord Blood Cells on Neonatal HI Brain Injury

**Figure 1:**
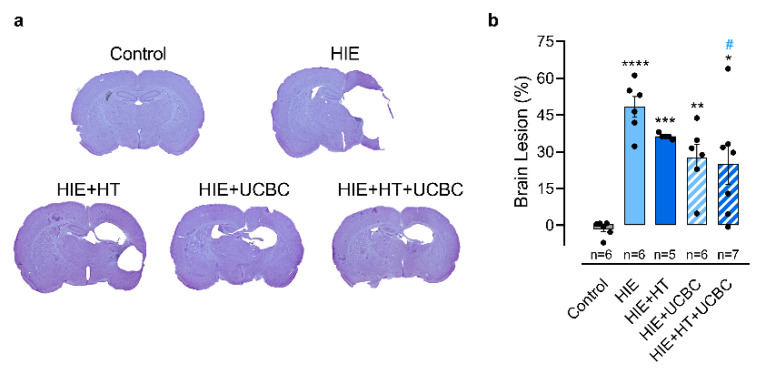
Intravenous administration of UCBC reduced the extension of the brain lesion evaluated 30 days after neonatal HI injury. (**a**) Representative images of brain sections stained with Cresyl Violet acquired at 5× magnification and (**b**) quantification of the brain lesion at P40, calculated as the ratio of the volume of the ipsilateral hemisphere to the contralateral hemisphere of 16 sequential sections. Data are expressed as mean ± SEM of five to seven animals per group (Table S5). Statistical analysis was performed using one-way ANOVA coupled with Tukey’s multiple comparison test (* *p* < 0.05, ** *p* < 0.01,*** *p* < 0.001, and **** *p* < 0.0001 compared to the control; ^#^ *p* < 0.05 compared to HIE; Table S16).

In order to evaluate the potential of UCBC for the treatment of neonatal HIE, HT alone (HIE + HT group), HT and UCBC (HIE + HT + UCBC group), or UCBC alone (HIE + UCBC group) were applied to newborn rat pups subjected to a severe HI insult. At P40, 30 days post neonatal HI injury (Figure 1), the HIE group presented a brain lesion affecting 48% of the ipsilateral hemisphere (*p* < 0.0001 vs. control group). There was no interaction between the effects of HT and UCBC (*p* = 0.452, Table S16). Moreover, HT did not affect the brain damage induced by the HI insult (*p* = 0.237), in contrast to UCBC (*p* = 0.015). Nonetheless, brain damage was only decreased in animals treated with HT+UCBC (*p* = 0.0294).

### 2.2. Impact of Hypothermia and Umbilical Cord Blood Cells on Glial Reactivity Triggered by Neonatal HI Brain Injury

**Figure 2:**
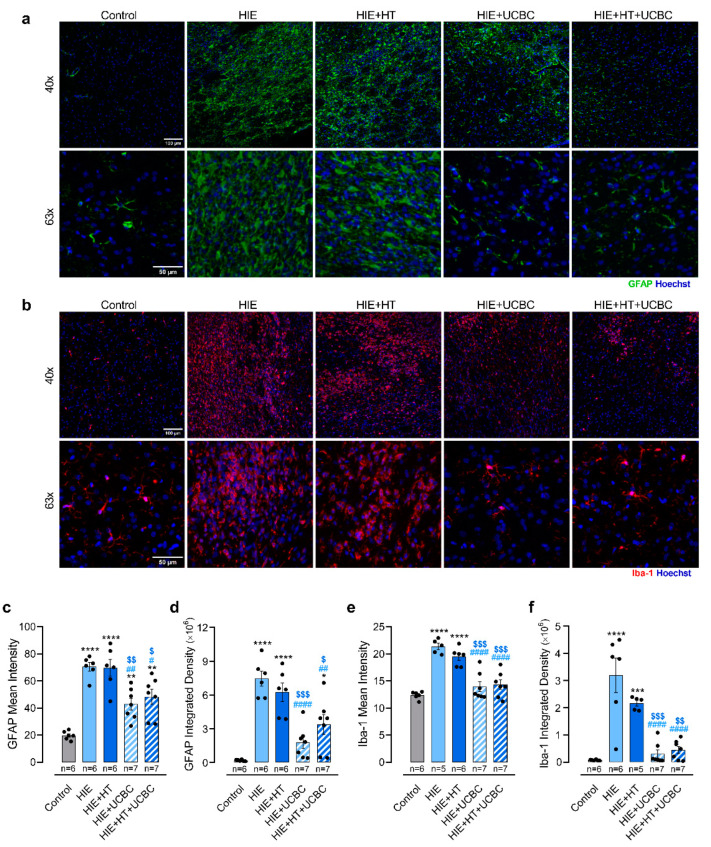
UCBC decreased GFAP and Iba-1 fluorescence intensity in the peri-infarct area 30 days after neonatal HI injury. Representative images of (**a**) GFAP (green) and (**b**) Iba-1 (red) immunostaining in the peri-infarct area were acquired at 40× and 63× magnification. Quantification of GFAP mean intensity (**c**), integrated density levels (**d**), Iba-1 mean intensity (**e**) and integrated density levels (**f**) at P40. The nuclei were counterstained with Hoechst 33342 (blue). Data are expressed as mean ± SEM of five to seven animals per group (Tables S6–S9). Statistical analysis was performed using one-way ANOVA coupled with Tukey’s multiple comparison test (* *p* < 0.05, ** *p* < 0.01, *** *p* < 0.001 and **** *p* < 0.0001 compared to the control; ^#^ *p* < 0.05, ^##^ *p* < 0.01, and ^####^ *p* < 0.0001 compared to HIE; ^$^ *p* < 0.05, ^$$^ *p* < 0.01, and ^$$$^ *p* < 0.001 compared to HIE + TH; Table S16).

Neonatal HI injury triggers a neuroinflammatory response that involves the long-term activation of glial cells, namely astrocytes and microglia [19]. To evaluate the impact of UCBC therapy on glial reactivity, GFAP and Iba-1 in the peri-infarct area were assessed by immunostaining (Figure 2a,b).

Neonatal HI injury increased GFAP mean intensity and integrated density levels in the peri-infarct area of lesioned animals (*p* < 0.0001, Figure 2c; *p* < 0.0001, Figure 2d), suggesting astrocyte reactivity and astrocyte recruitment or proliferation in the affected brain region. These changes were not affected by HT alone (Figure 2c,d). A decrease of approximately 31% in GFAP mean intensity (*p* = 0.0150) and 55% in GFAP integrated density (*p* < 0.0001) was observed in the HIE + HT + UCBC group and of approximately 39% (*p* = 0.0017) and 76% (*p* < 0.0001) in the HIE + UCBC group, respectively, when compared to the HIE group. There was no interaction between the effects of HT and UCBC on these parameters (*p* = 0.530 and *p* = 0.072, Table S16). Moreover, HT did not affect GFAP mean intensity (*p* = 0.649) or integrated density (*p* = 0.803), in contrast to UCBC (*p* < 0.0001 and *p* < 0.0001, respectively). These results suggest that UCBC isolated or combined with HT reduced astrocyte reactivity in this model.

Regarding microglia, there was an increase in the mean intensity and integrated density of Iba-1 labeling in the HIE group when compared with control (*p* < 0.0001, Figure 2e; *p* < 0.0001, Figure 2f), suggesting microglial reactivity and migration or proliferation of these cells to the peri-infarct area. This pattern was not affected by HT alone (Figure 2e,f). However, Iba-1 mean intensity and integrated density was decreased in groups treated with UCBC when compared with the HIE group (*p* < 0.0001 and *p* < 0.0001). There was no interaction between the effects of HT and UCBC on these parameters (*p* = 0.282 and *p* = 0.276, Table S16). Moreover, HT did not affect Iba-1 mean intensity (*p* = 0.551) or integrated density (*p* = 0.479), contrary to UCBC (*p* < 0.0001 and *p* < 0.0001, respectively). These data suggest that HT did not potentiate the effects of UCBC in decreasing astrocyte and microglial reactivity after neonatal HI injury.

### 2.3. Impact of Hypothermia and Umbilical Cord Blood Cells in the Sensorimotor and Motor Deficits Caused by Neonatal HI Brain Injury

After HIE, affected neonates can present long-term motor and cognitive deficits that deeply affect their health-related quality of life [20,21,22,23]. To determine if the positive impact of UCBC on brain lesion extension and glial activation led to improved functional outcomes, sensorimotor, motor, and cognitive skills were assessed at different neurodevelopmental stages.

**Figure 3:**
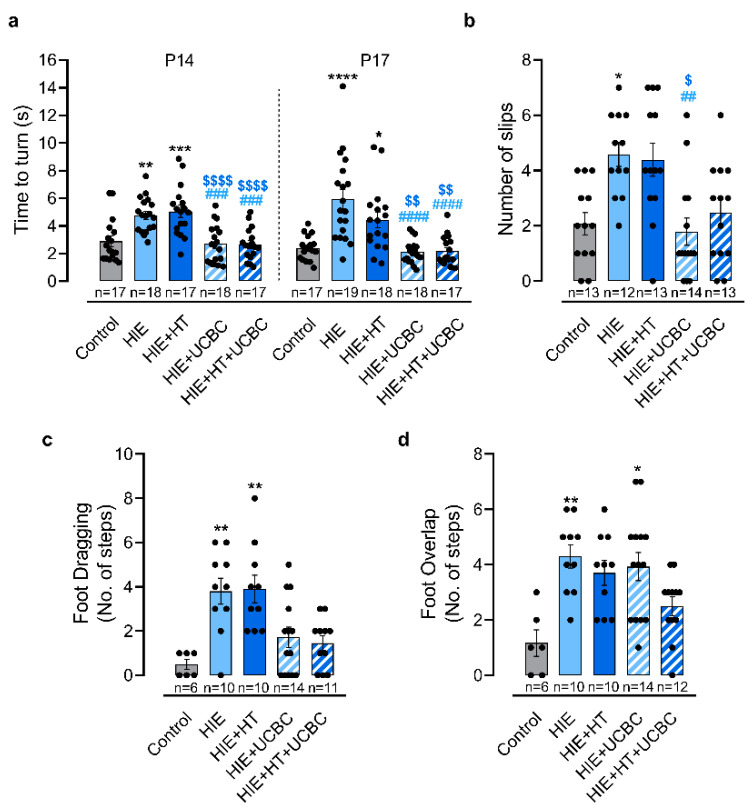
Intravenous administration of UCBC improved sensorimotor and motor impairments observed after neonatal HI injury at different stages of development. (**a**) Latency, in seconds, for the pups to rotate 180° to face uphill after release on a slanted slope in the negative geotaxis reflex test at P14 and P17. (**b**) Total number of limb slips in the ladder rung walking test at P30. The footprint test was used to determine foot dragging (**c**) and foot overlap (**d**) at P28. Data are presented as mean ± SEM of 17 to 19 animals per group in the negative geotaxis reflex test (Table S10); 12 to 14 animals per group in the ladder rung walking test (Table S11); and 6 to 14 animals per group in the footprint test (Tables S12 and S13). Statistical analysis was performed using one-way ANOVA coupled with Tukey’s multiple comparison test in the negative geotaxis reflex test and using the Kruskal–Wallis test coupled with Dunn’s multiple comparison correction test in the ladder rung walking test and footprint test (* *p* < 0.05, ** *p* < 0.01, *** *p* < 0.001, and **** *p* < 0.0001 compared to the control; ^##^ *p* < 0.01, ^###^ *p* < 0.001, and ^####^ *p* < 0.0001 compared to HIE; ^$^ *p* < 0.05, ^$$^ *p* < 0.01, and ^$$$$^ *p* < 0.0001 compared to HIE + TH; Table S16).

The sensorimotor function was assessed in early developmental stages by the negative geotaxis test. The results showed that pups in the HIE group took on average 4.78 ± 0.29 s to face uphill at P14 and 5.93 ± 0.71 s at P17, with longer times than the control group, which took an average of 2.90 ± 0.38 s at P14 and 2.39 ± 0.21 s at P17 to face uphill (*p* = 0.0022 at P14, *p* < 0.0001 at P17; Figure 3a). Additionally, this parameter was still impaired in the HIE + HT group when compared with controls (*p* = 0.0004 at P14, *p* = 0.0200 at P17) and with no significant difference from the HIE group. Sensorimotor impairment was decreased at both time points in groups that received UCBC treatment, alone (*p* = 0.0005 at P14, *p* < 0.0001 at P17) or in combination with HT (*p* = 0.0004 at P14, *p* < 0.0001 at P17), when compared with the HIE group. No interaction was identified between the effects of HT and UCBC on this sensorimotor test at both timepoints (*p* = 0.6116 and *p* = 0.1296 Table S16). Moreover, HT did not have an effect on the pups’ negative geotaxis reflex at P14 (*p* = 0.7467) and at P17 (*p* = 0.1450), unlike UCBC (*p* < 0.0001 and *p* < 0.0001, respectively). These results suggest that UCBC alone or combined with HT promoted a recovery of sensorimotor function at this neurodevelopmental stage.

Motor function was assessed using the footprint analysis at P28 and the ladder rung walking test at P30 being used for balance and coordination. Our data showed that HIE animals presented twice as much slips as controls, which indicates impaired locomotor function, i.e., the control group had an average of 2.1 slips and the HIE group had an average of 4.6 slips (*p* = 0.0282; Figure 3b). Once again, HIE rats treated with HT had similar outcomes to HIE rats, suggesting that HT alone was not able to rescue locomotor deficits (Figure 3b). However, HIE rats treated with UCBC alone or combined with HT presented a similar number of slips to controls (*p* > 0.9999 and *p* > 0.9999, respectively; Figure 3b), suggesting a moderate improvement in balance and coordination.

Data extracted from the footprint analysis revealed that animals from the HIE and HIE + HT groups had increased foot dragging, with an average of 3.80 and 3.90 dragged footprints, respectively, versus control animals which had an average of 0.5 (*p* = 0.0068 and *p* = 0.0068, respectively; Figure 3c). Regarding foot overlap, another parameter measured in the footprint analysis, animals in the HIE group presented increased foot overlap (*p* = 0.0051; Figure 3d), with HT alone not inducing rescue when compared to the HIE group. Treatment with UCBC alleviated the locomotor impairments identified in the HIE group, with HIE + UCBC and HIE + HT + UCBC groups presenting a number of steps with foot dragging not significantly different from controls (*p* > 0.9999 and *p* > 0.9999, respectively; Figure 3c).

### 2.4. Impact of Hypothermia and Umbilical Cord Blood Cells in the Cognitive Deficits Caused by Neonatal HI Brain Injury

**Figure 4:**
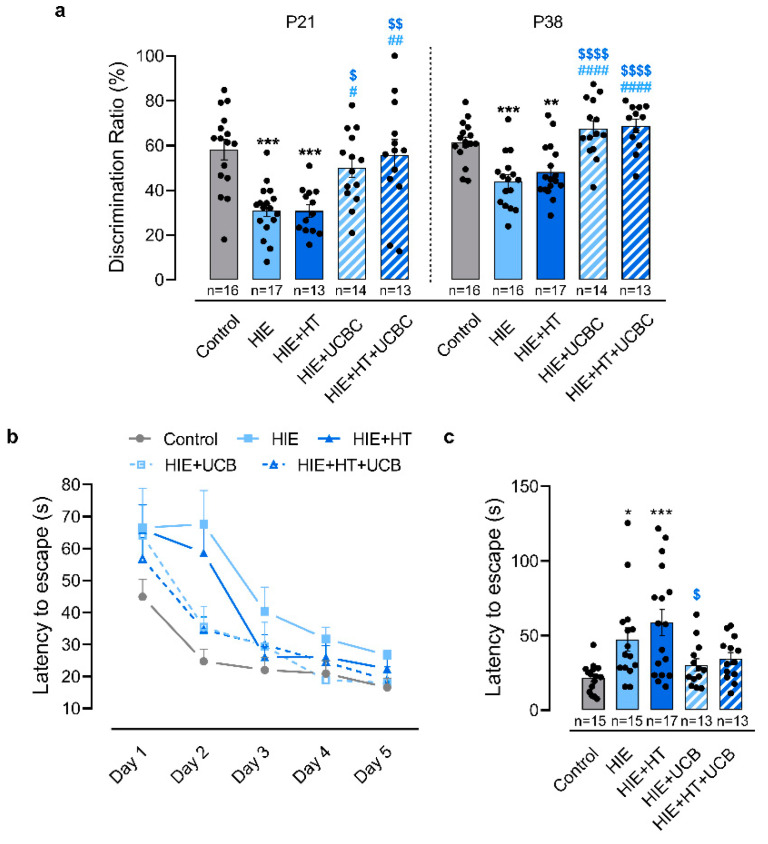
Intravenous administration of UCBC improved cognitive deficits after neonatal HI injury at different developmental stages. (**a**) Discrimination ratio, i.e., percentage of time spent exploring the novel object, in the novel object recognition test at P21 and P38. Latency, in seconds, to find the escape hole during the Barnes maze test throughout the five days of testing (**b**) and on the second day (**c**). Data are presented as mean ± SEM of 12 to 17 animals per group in the novel object recognition test (Table S14); and 13 to 17 animals per group in the Barnes maze test (Table S15). Statistical analysis was performed using one-way ANOVA coupled with Tukey’s multiple comparison test (* *p* < 0.05, ** *p* < 0.01, and *** *p* < 0.001 compared to the control; ^#^ *p* < 0.05, ^##^ *p* < 0.01, and ^####^ *p* < 0.0001 compared to HIE; ^$^ *p* < 0.05, ^$$^ *p* < 0.01, and ^$$$$^ *p* < 0.0001 compared to HIE + TH; Table S16).

Cognitive function was also assessed at different stages of development. Recognition memory, assessed by the novel object recognition test (Figure 4a), was impaired in the HIE group at P21 and P38, since these animals only spent about 31% and 44% of the time exploring the novel object, whereas control animals spent about 60% of the time on it at both time points (*p* = 0.0002 at P21, *p* = 0.0003 at P38; Figure 4a). Additionally, similarly to the motor function tests, HT alone was not sufficient to improve these outcomes. At both time points, the exploration time of the novel object by the animals in this group was inferior to that registered in the control group (*p* = 0.0004 at P21, *p* = 0.007 at P38) and no difference was observed to the HIE group. Both groups treated with UCBC improved their score in this test, spending more time exploring the novel object at both time points when compared with HIE (HIE + UCBC: *p* = 0.0143 at P21, *p* < 0.0001 at P38; HIE + HT + UCBC: *p* = 0.0012 at P21, *p* < 0.0001 at P38) and HIE + HT groups (HIE + UCBC: *p* = 0.0190 at P21, *p* < 0.0001 at P38; HIE + HT + UCBC: *p* = 0.0021 at P21, *p* < 0.0001 at P38). There was no interaction between the effects of HT and UCBC on recognition memory, assessed by the novel object recognition test, at both timepoints (*p* = 0.4746 and *p* = 0.6191 Table S16). Moreover, HT did not affect the animals’ exploration time of the novel object at P21 (*p* = 0.5304) and at P38 (*p* = 0.3929), as opposed to UCBC (*p* < 0.0001 and *p* < 0.0001, respectively). Thus, these results support that UCBC therapy, either alone or combined with HT, promoted a recovery of recognition memory which was sustained in the long-term.

Finally, spatial memory and learning were evaluated using the Barnes maze test (Figure 4b,c). The animals from the HIE and HIE + HT groups required more than twice the time to find the escape hole on the second day of testing compared to the control group (*p* = 0.0378 and *p* = 0.0005, respectively, Figure 4c). However, HIE rats treated with UCBC or HT + UCBC took a similar amount of time to find the escape hole on the second day as the control animals (Figure 4b,c). Moreover, there was no interaction between the effects of HT and UCBC on the latency to escape in the second day of the Barnes maze test (*p* = 0.6255). Furthermore, only UCBC affected the animals’ latency to escape (*p* = 0.0052).

Altogether, the results from the behavioral analysis show that UCBC-treated animals presented a total recovery of sensorimotor, motor, and cognitive functions to control levels, contrary to animals treated with HT alone. Additionally, the results from behavioral analysis suggest that the HT protocol used in this study did not enhance the beneficial effects of UCBC, since the HIE + HT + UCBC group had similar outcomes to the HIE + UCBC group.

## 3. Discussion

Neonatal HIE can cause severe neurodevelopmental impairment that may not be completely prevented by therapeutic hypothermia, the current standard of care [1]. Administration of UCBC has come up as a possible coadjuvant therapy for HIE, but scarce preclinical studies evaluated the combination of hypothermia with UCBC [15], and previous reports showed that a single dose of UCBC had a limited efficacy [11,13]. In the present study, three doses of UCBC were administered at 24, 48, and 72 h after an hypoxic-ischemic insult to target the secondary phase of HI injury in the developing brain. Most of the damage occurs during the secondary phase, which can last 6 h to 3 days in humans and is characterized by the acute upregulation of inflammatory pathways, apoptosis, mitochondrial failure, and breakdown of the blood–brain barrier [3]. By targeting this phase of injury, we aimed to take advantage of the reported anti-inflammatory [13] and pro-angiogenic [24] properties of the UCBC, and also to intervene in the progression to the tertiary phase of injury that is characterized by remodeling, chronic neuroinflammation, and astrogliosis [3]. Indeed, the results obtained with the present study show that the administration of three doses of UCBC, alone or combined with HT, was able to reduce the glial reactivity during the tertiary phase of injury. More importantly, this effect was consistent with improved sensorimotor, motor, and cognitive outcomes, which persisted in later stages of neurodevelopment. These results were observed in groups treated with UCBC alone or combined with HT, obtaining the same degree of improvement, thus suggesting that UCBC were responsible for the observed effects.

In the present study, unilateral severe HI injury was induced in P10 rats that caused the loss of about 48% of the ipsilateral hemisphere, mainly affecting the cortex, striatum, and hippocampus (Figure 1). This protocol aimed to mimic term neonatal HIE, since neurodevelopment of P10 rats is equivalent to the term human newborn [25]. Our results showed that 4 h of HT did not attenuate brain lesion of animals subjected to neonatal HI injury like others have previously reported [16,17,26]. However, the administration of UCBC combined with HT reduced brain lesion extension (Figure 1), in line with previous studies that administered single [8,13] or multiple doses of UCBC [9].

Previous reports have shown that inflammation strongly contributes to the neuropathological cascade associated with HIE. This complex response, which includes the activation of astrocytes and microglia as well as the infiltration of peripheral immune cells, is initiated immediately after the HI insult and can last for months or years after the injury [27,28]. In agreement with this, the results obtained in this study show that animals with HI injury presented a chronic increase in GFAP and Iba-1 levels in the peri-infarct area (Figure 2), which suggests an increase in astrocyte and microglia reactivity, that was not affected by HT alone. These results are in the same line of evidence of previous studies in which HT was not able to decrease glial reactivity [16,17]. Although another study reported that HT reduced microglial activation, in this previous study, animals were exposed to one hour of hypoxia, which resulted in a less severe injury [18]. Preclinical studies showed that the activation of astrocytes and microglia occurring in the early and late stages of HIE can be modulated by the administration of UCBC [7,9,10,13]. Indeed, in the present study, UCBC therapy, either in combination with HT or not, reduced GFAP and Iba-1 labeling, suggesting a blockage of glial reactivity (Figure 2). Once more, contrary to what was observed in previous studies that used MSC [16,17], the combination of HT with UCBC did not present an advantage when compared with the administration of UCBC alone. This difference might be related with the presence of several cell types in the umbilical cord blood instead of an enriched population of MSC or the effects of cell manipulations prior to administration. Nonetheless, increasing evidence suggests that HI insults in the developing brain result in a complex temporal and regional pattern of microglia activation that goes beyond the M1/M2 concept [29]. Most of the studies, including ours, evaluate microglial activation after stem cell therapy in HIE in vivo models using a single marker or focusing on limited time points, which is insufficient considering this complex response (reviewed by [29]). Thus, to unveil how stem cell therapy interferes with these processes, it will be necessary to use several markers of classical and alternative microglia activation at different stages of brain injury, in future studies.

Children who have suffered neonatal HIE can present decreased sensorimotor function, memory, cognitive and learning difficulties, and diminished motor capability, including locomotor and reduced fine motor skills compared with healthy peers [20,21,22,23]. Although the reduction of brain lesions and neuroinflammation are good indicators of recovery, inducing long-term functional improvement is especially important for the clinical translation of the therapies used. Thus, in this study, these functional outcomes were evaluated using a set of behavioral tests that are sensitive to identify sensorimotor, gait, memory, and learning alterations in the HIE rodent model across development [30]. While some preclinical studies evaluated the impact of UCBC on the functional deficits observed in HIE models, most of them used a reduced number of behavioral tests or focused on a limited developmental time window [7,11,13,31,32]. To address this, behavioral tests were implemented in this study during a period that comprises the neurodevelopment equivalent to human first infancy (P21) and late human adolescence/early adulthood (P38) [33]. Previous studies showed that administration of UCBC lessened short- and long-term behavioral deficits induced by HI lesions in the developing brain, specifically memory and learning impairments [9,12,18], gait alterations [10], and sensorimotor disability [7,13]. In this study, sensorimotor, motor, memory and learning impairments were observed after neonatal HI injury that persisted until early adulthood (Figure 3 and Figure 4). Although 4 h of HT did not attenuate these impairments, animals treated with UCBC, combined or not with HT, presented a total recovery of sensorimotor, motor, and memory function at early and later stages of injury (Figure 4). Once again, no difference was found between the animals treated with UCBC alone or with the combined therapies. Since UCBC therapy only partially prevented brain tissue loss, these functional improvements are most likely related to neuropathological changes, namely the reduction of glial reactivity triggered by the cells present in the umbilical cord blood (Figure 2). Indeed, previous studies correlated decreased neuroinflammation induced by UCBC treatment with reduced behavioral impairments in HIE rodent models [7,9,10,12,13,14]. Additionally, UCBC secrete a wide range of trophic factors, such as VEGF, BDNF, chemokines, among others [34,35], that contribute to neuronal repair, decreased inflammatory response, and promote brain plasticity, which may also mediate the functional recovery observed here.

The results show that the HT protocol used in the present study was not sufficient to ameliorate the functional deficits observed after a severe HI lesion in the developing brain. Herein, the period of HT was shorter (4 h) than the period used in the standard of care for term neonates diagnosed with HIE (72 h), which might explain this lack of effect. The HT protocol used in this study was previously described by others to induce neuroprotection in the Rice–Vannucci animal model for HIE [36,37]. For instance, Patel et al. (2015) reported neuroprotection in animals treated with 4 h of HT, even in those presenting severe HI lesion, but this neuroprotection was only translated into improved motor function in animals presenting a moderate HI lesion [37]. In this study, the neonatal rats were subjected to a longer period of exposure to hypoxia (90 min) than in the studies performed by Burnsed et al. (45 min) and Patel et al. (65 min), resulting in a more severe brain lesion [36,37]. Nonetheless, other studies showed that even 24 and 48 h of HT were insufficient to induce recovery in animals that presented a severe HI brain lesion [16,17]. Moreover, a recent meta-analysis of studies performed on the Rice–Vannucci model for HIE demonstrated that there is significant variability in the neuroprotection offered after 5 h of hypothermia and that a sex-dependent effect may occur [38]. Importantly, in a meta-analysis of studies with neonates diagnosed with HIE at term, it was found that, although therapeutic hypothermia reduced the overall mortality and neurodevelopmental disabilities by 15%, this therapy did not improve major neurodevelopmental disabilities and neuromotor delays in infants diagnosed with severe HIE [1].

As previously mentioned, in the present study, the combination of HT with UCBC was not advantageous in comparison with UCBC alone. These results might be explained by the higher dose of UCBC that was administered to the rat pups eliciting a maximal recovery, despite being within the range of doses previously used [8,9,39]. Thus, future studies using lower doses of UCBC might be necessary to assess if both therapies have a synergistic effect. Nonetheless, no adverse effects in using the combined therapies were observed in this study, recapitulating the safety studies already performed on neonates diagnosed with HIE [5,6].

In summary, the obtained results showed that UCBC administered during the secondary phase of neonatal HI injury combined with HT reduced brain damage, glial reactivity, and improved functional outcomes. Moreover, lesioned animals treated with UCBC alone had similar performance to animals treated with both therapies, suggesting the potential of UCBC therapy for patients that cannot undergo therapeutic hypothermia. These results demonstrate that UCBC therapy, combined or not with HT, has the potential to become part of the therapeutic approach to improve the prognosis of neonates who have suffered HIE.

## 4. Materials and Methods

### 4.1. Ethical Approval

Animal studies received ethical approval by the Ethical Committee of the University of Beira Interior and legal authorization by the competent Portuguese authorities (DGAV, 0421/000/000/2019) in respect of the European Directive for the protection of laboratory animals used for scientific purposes (2010/63/EU). The use of human stem cells received ethical approval from the Ethical Committee of the Faculty of Medicine of the University of Coimbra (075-CE-2019).

### 4.2. Animals

Animal studies were carried out in the animal facility of the Faculty of Health Sciences, University of Beira Interior (Covilhã, Portugal). Animals enrolled in this study were maintained in alternating 12 h light/dark cycles, rooming with their dam until weaning at postnatal day 21 (P21) and handled daily after the induction of the neonatal HI injury to monitor their wellbeing. The exact number of animals used can be found in Tables S1 and S2. The experimental unit for each procedure is the individual animal, each independently allocated to a treatment group (HT, UCBC, or HT + UCBC).

### 4.3. Induction of Neonatal Hypoxic-Ischemic Brain Injury

Unilateral HI brain lesion was induced in male and female Wistar rat pups at P10 according to the Rice–Vannucci model for HIE [40]. Animals were weighted (Table S4) and anesthetized with isoflurane (5% for induction, 1.5–2% for maintenance; Isoflo, Zoetis), and a 1 cm incision was made in the midline of the pup’s neck to isolate and ligate the left common carotid artery (CCA) with 6−0 silk suture (F.S.T). One hour later, animals were placed for 90 min in an airtight chamber filled with 8% oxygen balanced with 92% nitrogen (Airliquide). Control animals were anesthetized, the CCA was isolated but not ligated, and were exposed to room air. The ambient temperature was optimized (34 °C) to maintain the pup’s internal temperature at 36–37 °C.

### 4.4. Hypothermia

Two hours after the induction of neonatal HI, rat pups to be subjected to HT were placed in a chamber kept at 27 °C to decrease the internal body temperature to 31–32 °C. The rat pups were maintained in hypothermia for 4 h [36,37]. The HT protocol was initially optimized by measuring the internal body temperature of three pups using a rectal probe (Table S3). To prevent the warming of the animals due to manipulation and to reduce the discomfort resulting from the introduction of the rectal probe, after the optimization of the HT conditions, the internal body temperature of the animals was only monitored at the middle and at the end of the procedure. Rat pups not subjected to HT were placed in a heated chamber to maintain their internal temperature at 36–37 °C (normothermia).

### 4.5. Preparation and Administration of UCBC

Human umbilical cord blood samples were obtained from Stemlab, S.A. (Cantanhede, Portugal). Briefly, to perform umbilical cord blood volume reduction, hydroxyethyl starch (HES) was added to the anticoagulated umbilical cord blood samples in a 1:3 ratio. The red blood cells were separated by gravity/sedimentation. The plasma and the buffy coat containing the white blood cells were separated from the red blood cells by centrifugation. In order to concentrate the buffy coat, the extra plasma was removed so that a final volume of 20 mL was obtained. Then, a DMSO-dextran solution was added to the volume-reduced umbilical cord blood samples, which, after a controlled-rate freezing, were stored in nitrogen tanks.

The cryopreserved human UCBC were administered 24, 48, and 72 h after neonatal HI injury. On the day of administration, the previously processed umbilical cord blood samples were thawed, centrifuged at 930× *g* for 10 min, and cellular density was determined using the Trypan Blue exclusion method.

For the administration, rat pups were anesthetized with isoflurane, and 10^6^ UCBC diluted in 200 µL of phosphate-buffered saline (PBS) were administered in the tail vein using a 29-gauge insulin syringe (Terumo). Rat pups from experimental groups without UCBC received an intravenous administration of 200 µL of PBS.

### 4.6. Behavioral Analysis

#### 4.6.1. Negative Geotaxis Reflex

The negative geotaxis reflex test was used to assess the rat’s motor coordination early in development, at P14 and P17. For this test, rat pups were placed downhill on a 45° slanted slope, and the time required for the pups to face uphill was recorded. No animals were excluded from the analysis.

#### 4.6.2. Novel Object Recognition Test

The novel object recognition test was used to evaluate recognition memory at two different stages of development, P21 equivalent to human infancy and P38 equivalent to human adolescence [33]. The day before testing, rats were familiarized with the empty testing arena for 10 min. On the testing day, rats were initially exposed to two identical objects for 10 min (familiarization phase), and after 30 min, rats were exposed to a familiar object and a novel object for 5 min (novel object exposure phase). To exclude possible bias, the familiar object replaced by the novel one was random. Both testing phases were recorded with a video camera using the ANY-Maze software (Stoelting). The time spent with the familiar object and the novel object was determined for each animal, and the percentage of time spent with the novel object was calculated using the formula: Discrimination Ration (%) = (time spent with novel object)/(total exploration time) × 100. Animals that had a total exploration time inferior to 10 s were excluded from the analysis.

#### 4.6.3. Footprint Test

The footprint test was used to identify locomotor deficits and gait abnormalities [41]. This test was taken at P28, which corresponds to early human childhood [33]. The fore and hind paws of the rats were dipped in non-toxic paint, and animals were encouraged to walk 100 cm along a narrow corridor covered with paper towards a black box to obtain the footprints. The footprint pattern was evaluated considering ten consecutive steps, and the number of steps presenting foot dragging or overlapping footprints was counted. Animals that were not motivated to cross the corridor were excluded from the analysis.

#### 4.6.4. Ladder Rung Walking Test

The ladder rung walking test was employed to assess motor function at P30, which is equivalent to human childhood [33]. The ladder rung apparatus consisted of a 100 cm long corridor, delimited by two transparent acrylic side walls, with metal rods (0.3 cm of diameter) inserted 1 cm apart. The rats were encouraged to walk through the apparatus four consecutive times, and all trials were recorded. Each video was examined in slow motion to count the number of paw slips between the rods. Animals that were not motivated to cross the ladder were excluded from the analysis.

#### 4.6.5. Barnes Maze Test

The Barnes maze test was applied to evaluate spatial memory and learning. The apparatus consisted of a round table with 23 holes, with one of the holes leading to an escape box. This apparatus was set in a well-lit room with four visual cues placed in a fixed position throughout all testing days. The testing began at P31, with the habituation phase followed by the first day of the acquisition phase. During habituation, the rat was placed inside the escape box for one minute and afterwards was allowed to explore the apparatus freely; if the rat failed to find the escape hole within five minutes, it was guided to it. After entering the escape box, the animal was maintained there for 30 s. The acquisition phase consisted of two trials per day, with an inter-trial interval of 20 min, for five consecutive days. For each trial, the rat was placed in the center of the apparatus and allowed to explore for a maximum period of three minutes. The test was recorded with a video camera using the ANY-Maze software (Stoelting).

### 4.7. Histology and Fluorescence Microscopy

Upon completion of the behavioral tests, animals were euthanized with an anesthetic overdose of 200 mg/kg Ketamine (Imalgène 1000, 100 mg/mL) and 10 mg/kg Xylazine (Rompun, 20 mg/mL), followed by cardiac perfusion with PBS and 4% paraformaldehyde (PFA). After perfusion, the brains were collected and immersed in 4% PFA for 16 h at 4 °C. Then, fixed brains were placed in 30% sucrose solution until sinking, frozen in liquid nitrogen, and stored at −80 °C until sectioning. Frozen brains were sectioned with a thickness of 40 µm on a cryostat (Leica CM3050) and sections were collected at 240 µm intervals.

#### 4.7.1. Cresyl Violet Staining

Brain lesion extension was assessed in sixteen sequential brain sections for each animal. Frozen brain sections were mounted in poly-lysin coated glass slides (VWR) and stained with 0.05% Cresyl Violet Acetate (Merck) using the Sakura TissueTek Automated DRS 2000 automated slide stainer. Images were acquired with the Axio Imager A1 Microscope (Zeiss) with a 5× objective (EC Plan-Neofluar 4×/0.16 M27), and the volume of brain tissue in the left (ipsilateral) and right (contralateral) hemispheres was determined with the Cavalieri’s Principle Probe of the StereoInvestigator software (MBF Bioscience, Williston, VT, USA). Brain lesion extension was calculated as (V_contralateral_ − V_ipsilateral_)/V_contralateral_ × 100.

#### 4.7.2. Immunohistochemistry

Four sequential brain sections per animal were selected for immunohistochemical analysis. Brain sections were permeabilized with PBS-1% Triton-X100 for 12.5 min and blocked for 2 h with 10% fetal bovine serum (Biochrom, London, UK). Then, sections were incubated for 72 h, at 4 °C, with a rabbit anti-glial fibrillary acidic protein (GFAP) antibody (DAKO Z0334, 1:200), or a rabbit anti-ionized calcium binding adaptor molecule 1 (Iba-1) antibody (WAKO 019-19741, 1:2000). Then, the sections were incubated for 2 h, at room temperature, with the secondary antibodies (anti-rabbit A488 Molecular Probes A11008, 1:1000 or anti-rabbit A546 Invitrogen A11010, 1:1000). Nuclei were stained with Hoechst 33342 (Invitrogen H1399, 1:1000). Images of the peri-infarct area were acquired using the LSM 710 AxioObserver Microscope (Zeiss, Jena, Germany) with a 40× objective (EC Plan-Neofluar 40×/1.3 Oil DIC M27) for the quantification of mean fluorescence intensity and integrated density, and a 63× objective (Plan-Apochromat 63×/1.4 Oil DIC M27) for the evaluation of glial cell morphology. Mean fluorescence intensity and integrated density were quantified with ImageJ software in four non-overlapping fields of view of the peri-infarct area (or equivalent region in the control group) per section.

### 4.8. Statistical Analysis

Statistical analysis was performed using GraphPad Prism v8.0.1 (GraphPad Software Inc., San Diego, CA, USA) and IBM SPSS Statistics for Windows v28.0.0.0 software. Outliers from each data set were identified by the Grubbs’ test (α = 0.05) and excluded. The Kruskal–Wallis test coupled with Dunn’s multiple comparison correction test, one-way ANOVA coupled with Tukey’s multiple comparison test, or two-way ANOVA were used according to the type of data. A *p*-value of 0.05 was considered for significance. Test values for each experiment and the source data used in the analysis are included in Tables S5–S16, in line with the FAIR Data Principles [42].

## Data Availability

Data supporting reported results can be found in the supplementary materials.

## Supplementary Materials

The following supporting information can be downloaded at: https://www.mdpi.com/article/10.3390/ijms24010257/s1.
